# Changes of PBP5 Gene Expression in Enterococcal Isolates from Renal Transplantation Recipients

**DOI:** 10.1155/2013/687156

**Published:** 2013-06-19

**Authors:** T. Jarzembowski, A. Daca, J. Witkowski, B. Rutkowski, J. Gołębiewska, A. Dębska-Ślizień

**Affiliations:** ^1^Department of Microbiology, Medical University of Gdańsk, Do Studzienki 38 Street, 80-227 Gdańsk, Poland; ^2^Department of Pathophysiology, Medical University of Gdańsk, Dębinki 7 Street, 80-211 Gdańsk, Poland; ^3^Department of Nephrology, Transplantology and Internal Medicine, Medical University of Gdańsk, Dębinki 7 Street, 80-952 Gdańsk, Poland

## Abstract

The aim of the study was to evaluate changes in expression of PBP5 gene associated with immunosuppression. A linear locked nucleic acid (LNA) probe was used to measure resistance gene expression by the Flow-FISH method. Expression of the PBP5 gene measured by Flow-FISH was higher in enterococcal strains isolated from renal transplantation (RTx) recipients than in commensal strains. Additionally, in contrast to commensal strains in isolates from RTx patients, PBP5 gene expression was 17.45% higher in biofilms than in planktonic cells. Detailed comparison also showed that cyclosporine seemed to induce higher expression of PBP5 as compared to tacrolimus.

## 1. Introduction

 There is growing evidence that *Enterococcus* spp., which are usually considered harmless commensal, can cause serious infections [1]. Among the other bacteria, enterococci have emerged as important etiologic agents of inflammation in renal transplantation (RTx) recipients. It is sometimes difficult to manage enterococcal infections due to common antibiotic resistance, heterogeneity of resistance, and modulation of resistance gene expression [2]. Enterococci are intrinsically resistant to the most frequently used antibiotics as *β*-lactams. The *β*-lactam-based antibiotics bind the transpeptidase domain of penicillin-binding proteins (PBPs), causing a loss of cell wall integrity. The intrinsic moderate resistance of *Enterococcal *spp. to *β*-lactam-based antibiotics results from the presence of a particular class PBP, which takes over the transpeptidase function of other PBPs when they are inhibited by antibiotics [3]. Such mechanism lets bacterial cell avoid the lethal action of drugs but usually results in the loss of the bacterial fitness to the host [4]. To minimize this effect, bacteria regulate expression of resistance genes in relation to environmental condition.

This aspect of mechanism of resistance makes studying enterococcal resistance to *β*-lactams difficult and in our opinion requires use of advanced method as flow cytometry. Because of analyzing many more cells than by conventional methods, rare cell types are more likely to be detected and the results are accessible to statistical analysis. By combining flow cytometry and FISH technique the Flow-FISH one may obtain excellent method for detection of gene expression [5].

The aim of this study was to analyze expression of PBP5 genes under immunosuppression in (RTx) recipient's isolates.

## 2. Materials and Methods

Forty enterococcal strains were isolated from urine and faeces of twenty RTx recipients with no current antibiotic therapy hospitalized at the Medical University of Gdansk. As reference group, seven commensal strains isolated from healthy volunteers and reference strains ATTC 51299 and ATTC 29212 were used in this study. The isolates were identified to species level by strep ID test (BioMerieux) and classified as different strains of *Enterococcus faecalis* by biochemical and resistance profiles. MIC value for penicillin was determined by *E*-test method. Biofilms of these strains were formed in flat-bottom wells (TRP, Switzerland). To analyze the effect of immunosuppression in vitro on bacterial cells, the strains were additionally cultured with serial dilutions of cyclosporine and tacrolimus starting from a therapeutic concentration of 2000 ng/mL and 200 ng/mL, respectively.

To evaluate gene expression by the Flow-FISH method we used a linear locked nucleic acid (LNA) probe, GACCACGCAAGAAGCAACAAGAGGG-5′FITC containing nucleic acid analogs with higher affinity for DNA and RNA [6]. Influence of immunosuppressant on background fluorescence of medical strains was excluded by comparison of fluorescence signal from bacteria incubated with ciclosporine and tacrolimus, respectively.

As a positive control, Enfl84 probe (3′-ACGTGAGTTAACCTTTCTCC) [7] targeting 16sRNA gene was used. Oligonucleotides were synthesized commercially (Metabion), labeled with fluorescein isothiocyanate (FITC) and tested for specificity against the set of reference organisms listed above. For hybridization, the procedure described by Waar et al. [7] was adopted and modified [8]. Briefly, cell membranes were permeabilized by incubation for 30 min at 37°C in permeabilization buffer (Tris-EDTA) consisting of 1 mg/mL lysozyme (DNA Gdańsk, Poland). Then, the cells were suspended in 1 mL of 0.9% NaCl and sonicated for 2 min on ice. To ensure permeabilisation of the cells, we used propidium iodide (PI, 1 *μ*g/mL) staining of DNA. Particles without PI fluorescence (FL3) were excluded from further investigation. Fluorescence of particles was determined using a FACScan flow cytometer (Becton-Dickinson, Franklin Lakes, NJ, USA). The mean probe fluorescence (FL1) normalized by DNA fluorescence (FL3) and the median fluorescence (MFL1) weighted by percentage of probe-binding particles (FL1 positives) were analyzed. Results were tested by analysis of variance (ANOVA) by StatSoft software (Statistica 10).

## 3. Results and Discussion

MIC for penicillin varied in studied strains from 1 *μ*g/mL up to 256 *μ*g/mL. With break point of 16 *μ*g/mL, majority of medical isolates were recognized resistant to penicillin in contrast to commensal strain where median MIC value was 1 *μ*g/mL.

**Figure 1:**
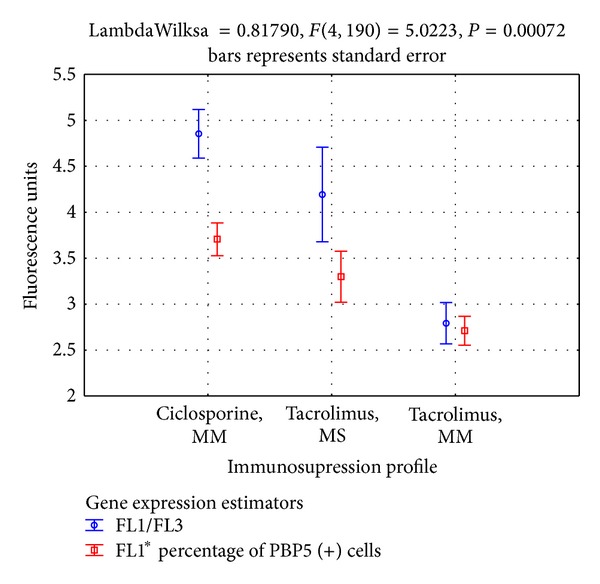
Comparison of PBP5 gene expression by Flow-FISH in *E. faecalis* isolates from RTx patients. MM: mycophenolate mofetil and MS: mycophenoate sodium.

Similar to the previous results, also Flow-FISH results showed that, in enterococcal strains isolated from RTx patients, the expression of the PBP5 gene was higher than in commensal strains (regardless of whether the strains were isolated from urine or faeces). The median values of PBP5 gene expression estimators were 0.48 (FL1/FL3) and 0.46 (MFL1*FL1 (+)) for commensal strains and 3.43 (FL1/FL3) and 3.09 (MFL1*FL1 (+)) for clinical strains, respectively. We also found out that PBP5 gene probe binding differs in relation to immunosuppressant used in therapy, According to analysis of Flow-FISH results, cyclosporine seemed to promote higher expression of PBP5 as compared to tacrolimus (Figure 1). Higher median MIC value for penicillin in isolates from patients treated with cyclosporine (265 *μ*g/mL) than in tacrolimus recipients (132 *μ*g/mL) also supports such findings, but this result was not proved by statistical tests. Calcineurin inhibitors tacrolimus and cyclosporine (CsA) are the mainstay of maintenance immunosuppression in renal transplant recipients. According to KDIGO Clinical Practice Guidelines for the Care of Kidney Transplant Recipients, tacrolimus is suggested as first-line calcineurin inhibitor. Compared to CsA, tacrolimus reduces the risk of acute rejection and improves graft survival during the first year of transplantation. However, there is no clear benefit of tacrolimus use in terms of patient mortality, incidence of malignancy, infection, delayed onset of graft function, or blood pressure. Both drugs also have distinct side-effects profiles, for example, with lower incidence of new-onset diabetes after transplantation in patients treated with CsA [9].

In our study, we have not found correlation between MIC value and PBP5 gene expression. However, increased expression of PBP5 is only rarely associated with high MIC value in clinical isolates [10]. According to recent research, it is possible that in other strains the PBP5 variants confer different PBP affinity, and, as a result, different binding to penicillin or posttranslational modification of PBP5 occurs [3, 10].

**Figure 2:**
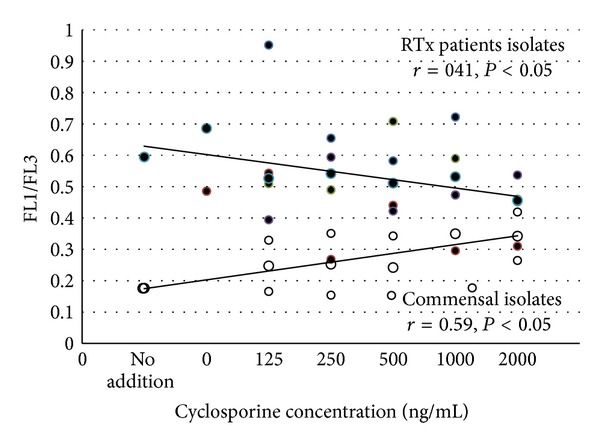
In vitro influence of ciclosporine on PBP5 expression in enterococcal biofilm. White points: results for commensal isolates and black points: values for RTx patient isolates.

**Figure 3:**
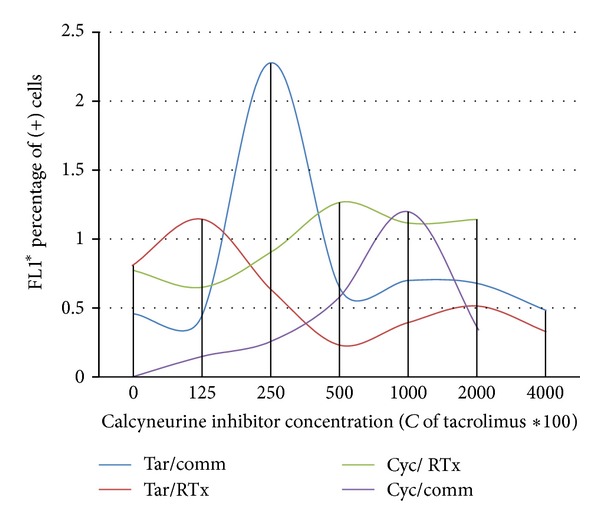
Comparison of changes of PBP5 expression in planktonic cells of *E. faecalis*. Comm.: commensal isolates, RTx: renal transplant recipients' isolates, Tar: tacrolimus, and Cyc: cyclosporine.

The results of in vitro study showed that higher expression of PBP5 is a result of the direct influence of the immunosuppressant on bacterial cells, rather than a result of immunomodulation within the host patient. In commensal strains, fluorescence of PBP5 gene probes increased with increasing concentration of cyclosporine, both in biofilm and planktonic cells of commensal strains (Figures 2 and 3). Surprisingly, in RTx isolates in vitro induction was observed only in planctonic cells, but in biofilm “basic” expression level was still much higher than in commensal strains (Figure 2). In contrast to cyclosporine, only low concentration of tacrolimus induces PBP expression (Figure 3).

Interesting differences were also noted during comparison of PBP5 gene expression in biofilm and planktonic cells. Assuming a lower rate of cell division in biofilms, it could be expected that PBP gene expression would be higher in planktonic cells. In fact, the PBP5 gene expression in commensal strains was 14.6% higher in plankton than in biofilms. In contrast to this observation, in isolates from RTx patients, PBP5 gene expression was 17.45% higher in biofilms than in plankton. Unfortunately, our knowledge of the role of PBP5 in cell wall biosynthesis remains insufficient [10, 11], so the interpretation of the above observation would remain speculative. 

On the other hand, observation of especially high PBP5 expression on biofilm suggests that ciclosporine and tacrolimus not only influence bacterial gene expression but may also modify biofilm properties. To analyze this effect of immunosuppressant, other studies including biofilm biomass and composition are needed.

To conclude, antibiotic therapy using *β*-lactam-based antibiotics may be more effective if tacrolimus, rather than cyclosporine, is chosen as immunosuppressant. However, further studies are still needed to analyze other aspects of the effect of cyclosporine on bacterial cells.
